# ADRA2A is involved in neuro-endocrine regulation of bone resorption

**DOI:** 10.1111/jcmm.12505

**Published:** 2015-03-27

**Authors:** Vid Mlakar, Simona Jurkovic Mlakar, Janja Zupan, Radko Komadina, Janez Prezelj, Janja Marc

**Affiliations:** a Department of Clinical Biochemistry, Faculty of Pharmacy, University of LjubljanaLjubljana, Slovenia; b Department for Research and Education, General and Teaching Hospital CeljeCelje, Slovenia; c Department of Endocrinology, Diabetes and Metabolic Diseases, University Medical Centre LjubljanaLjubljana, Slovenia

**Keywords:** ADRA2A, osteoporosis, bone, resorption, osteoblast, adrenergic signalling

## Abstract

Adrenergic stimulation is important for osteoclast differentiation and bone resorption. Previous research shows that this happens through β2-adrenergic receptor (AR), but there are conflicting evidence on presence and role of α2A-AR in bone. The aim of this study was to investigate the presence of α2A-AR and its involvement in neuro-endocrine signalling of bone remodelling in humans. Real-time polymerase chain reaction (PCR) and immunohistochemistry were used to investigate α2A-AR receptor presence and localization in bone cells. Functionality of rs553668 and rs1800544 single nucleotide polymorphism SNPs located in α2A-AR gene was analysed by qPCR expression on bone samples and luciferase reporter assay in human osteosarcoma HOS cells. Using real-time PCR, genetic association study between rs553668 A>G and rs1800544 C>G SNPs and major bone markers was performed on 661 Slovenian patients with osteoporosis. α2A-AR is expressed in osteoblasts and lining cells but not in osteocytes. SNP rs553668 has a significant influence on α2A-AR mRNA level in human bone samples through the stability of mRNA. α2A-AR gene locus associates with important bone remodelling markers (BMD, CTX, Cathepsin K and pOC). The results of this study are providing comprehensive new evidence that α2A-AR is involved in neuro-endocrine signalling of bone turnover and development of osteoporosis. As shown by our results the neurological signalling is mediated through osteoblasts and result in bone resorption. Genetic study showed association of SNPs in α2A-AR gene locus with bone remodelling markers, identifying the individuals with higher risk of development of osteoporosis.

## Introduction

Osteoporosis (OP) has a complex genetic architecture because of numerous underlying molecular processes and genes involved in bone metabolism. Each mechanism is contributing only little to the bone turnover ratio which is a reason why only little variability in BMD can be explained 1,2. Our previous research showed that adrenergic receptor α2A (α2A-AR) is up-regulated in osteoporotic bone osteoblasts relative to osteoarthritic bone osteoblasts, suggesting that this type of AR might play an important role in the development of OP 3. It has been known for some time that AR stimulation results in osteoclast differentiation, which leads, in turn, to bone resorption 4. There is substantial evidence to suggest that this happens through β2-AR signalling 5–10, since this should be the only AR expressed by osteoblasts 6,8. Further studies have shown that beta blockers and agonists have opposite effects on bone mass in adult animals 9,11,12. In contrast to above research, recent publication on mice shows that it is not only β2-AR that plays a role in bone resorption but that the same role is also performed by α2A-AR and α2C-AR. Fonseca *et al*. showed that double adrenoreceptor knockout (ARKO) female mice present a high bone mass phenotype 13. Moreover, it has also been shown that ARKO mice exhibit lower tartrate-resistant acid phosphatase and receptor activator of NF-kB 13. The α2A-AR polymorphism 1780G>A (rs553668) was found to be associated with numerous different phenotypes, glucose levels 14, insulin levels and the development of type 2 diabetes 15, obesity and body fat percentage 16, responsiveness to stress 17, endurance status 18, platelet function 19–21 and blood pressure 22. The precise mechanism of its action is unknown; however, constructs of luciferase reporter and the α2A-AR 3′ UTR region where polymorphism is located have shown that it probably affects mRNA levels through mRNA stability 23. Another polymorphism -1291C>G (rs1800544) also showed association with different phenotypes such as attention-deficit/hyperactivity disorder inattentive type 24, tobacco smoking 25, adolescent personality 26, sweet food product consumption 27 and olanzapine treatment 28. The aim of this study was to investigate whether α2A-AR has a role in human neuro regulation of bone remodelling. In the study, we first addressed the issue of α2A-AR expression in human bone samples using immunohistochemistry and real-time polymerase chain reaction (PCR) to resolve dilemma of α2A-AR presence in bone. Next, above described polymorphisms (rs553668 and rs1800544) were tested for functionality to find suitable genetic markers of α2A-AR gene locus. Next, genetic association study was performed to investigate α2A-AR association with OP and bone turnover markers.

## Material and methods

### Immunohistochemistry

Bone samples of intertrochanteric trabecular bone from 27 osteoporotic patients with femoral neck fracture (OP patients), as well as from 21 patients with osteoarthritis (OA) and 14 sex and age-matched autopsy participants (C) as controls including only those where the medical history did not include any disorders affecting bone were obtained (Table1). Samples were fixed in neutral buffered formalin for a maximum of 24 hrs and decalcified with EDTA. In the cases of the autopsy participants, cross-sections from the femoral head were also collected. Paraffin sections were prepared *via* a routine procedure at the University of Ljubljana’s Institute of Pathology. Immunohistochemistry was performed on the four samples that showed the highest (OP patient), an intermediate (OA patient) and the lowest (one autopsy participant and one OP patient) gene expression of α2A-AR, respectively. In addition, immunostaining was carried out on one cross-section of the femoral head from the autopsy participant as well. Paraffin sections were dewaxed, rehydrated and microwaved for 10 min. in 0.01 M sodium citrate buffer (pH 6) to release masked epitopes. The immunohistochemistry was hereinafter performed with a Mouse and Rabbit specific HRP/DAB detection IHC kit (ab64264; Abcam, Cambridge, UK) in accordance with the manufacturer’s procedures. Tris buffered saline (TBS), with the addition of Triton X-100 (Sigma-Aldrich, Steinheim, Germany), was used for washing purposes throughout the whole procedure. The primary rabbit polyclonal antibody to α2A-AR (ab65833; Abcam) was diluted 1:100 in TBS buffer and all control slides of each sample were treated with TBS only (negative controls). All slides were incubated overnight at 4°C in a humidified chamber. The specificity of the antibody used had been previously verified in our laboratory on HOS cell culture (data not shown). The tissue sections were counterstained with haematoxylin solution (Thermo Shandon, Pittsburgh, PA, USA) and examined with an Olympus BX50 microscope (Olympus, Hamburg, Germany). The intensity of staining in osteoblasts, lining cells and osteocytes was compared across all samples by two independent, blinded evaluators. Images were acquired using an Olympus XC50 camera and the CellSens Dimension program 1.6.0 (Olympus).

**Table 1:** Profile of patients used for α2A-AR gene expression analysis

Number of patients	Female (88)/Male (52)
Age (years)	71.95 ± 10.64/67.00 ± 11.46
Height (cm)	162.2 ± 6.4/172.7 ± 7.9
Weight (kg)	71.6 ± 12.2/80.1 ± 13.6
Osteoporotic samples (*N*)	40/11
Osteoarthrotic samples (*N*)	45/30
Controls (*N*)	3/11
Gene expression_TOT_ (mean ± SD)	1.283 ± 0.160 (74)/1.258 ± 0.150 (39)
Gene expression_OP_ (mean ± SD)	1.291 ± 0.199 (49)
Gene expression_OA_ (mean ± SD)	1.265 ± 0.096 (52)
	

OP, osteoporosis; OA, osteoarthritis; C, authopsy.

### Gene expression association study

The expression of the α2A-AR gene was analysed by quantitative real-time polymerase chain reaction (qPCR) assay in bone samples of Slovenian patients undergoing either hemiarthroplasty or total hip arthroplasty as a consequence of low-energy femoral neck fracture (51 OP patients) or primary hip OA (75 OA patients). Bone tissue samples obtained from autopsy without any diagnosed systemic disease were used as controls (14 C subjects). Patients were included in the study in a consecutive manner over a period of 1.5 years as they were directed to arthroplasty at the Department of Traumatology in the General and Teaching Hospital Celje as a result of receiving a diagnosis of OP or OA. OP was diagnosed by radiologically confirmed low-energy femoral neck fracture and WHO criteria. The diagnosis of OA was established by clinical and radiographic criteria according to the Harris hip score 29. All OP patients were submitted to arthroplasty within 24 hrs following femoral neck fracture. Bone tissue samples (approximately 1 cm^3^) were collected during surgical procedures of femoral osteotomy from the trabecular bone at the metaphyseal cutting plane. A proportion of the bone samples was immediately frozen in liquid nitrogen and stored at −80°C until RNA and DNA extraction. The exclusion criteria for the enrolment of OP and OA patients, as applied through the questionnaire, laboratory results and interview, included the following: secondary OP or OA, liver and kidney diseases, endocrinological disorders and medical anamnesis on receiving medications with a known influence on bone metabolism. The study was approved by the Ethical Committee of Republic of Slovenia and all patients gave written informed consent.

DNA and RNA were extracted using a TRI reagent (Sigma-Aldrich Chemie) in accordance with the manufacturer’s procedure. DNA and RNA concentration and purity were evaluated on a ND-1000 (Thermo Scientific, Wilmington, DE, USA). With respect to RNA, the RNA integrity number (RIN) was evaluated using a Bioanalyzer 2100 (Agilent, Santa Clara, CA, USA; for 50 samples, mean RIN = 5.7; range, 2.4–8.4). Where the RIN was very low, the extraction of RNA was repeated and confirmed by real-time analysis. For reverse transcription, the High Capacity RNA-to-cDNA Kit (Applied Biosystems, Foster, CA, USA) was used. Complementary DNA (cDNA) from the total RNA extracted was synthesized according to our previously described procedure 30 and stored at −80°C until the measurement of gene expression. Gene-specific primers for α2A-AR (forward: 5′-CTGCGTTTCCTCGTCTGG-3′, reverse: 5′-CAGCAAGAAGCTTCCCTTGT-3′) and 5× HOT FIREPol EvaGreen qPCR Mix Plus (Solis, BioDyne, Tartu, Estonia) were used. The reaction was performed on a LightCycler 480 (Roche Diagnostics, Rotkreuz, Switzerland) under the following conditions: 1 cycle of 95°C for 15 min., followed by 45 cycles of 15 sec. at 95°C, 20 sec. at 59°C and 20 sec. at 72°C. At the end, the generation of the melting curve was performed starting with 95°C for 5 sec. and followed by increasing the temperature from 55 to 95°C at a heating rate of 0.02°C/sec. All samples were quantified in triplicates. Dilution series of cDNA were prepared to create a relative standard curve with each run and absolute quantification of the data was performed with the second derivative maximum method (LightCycler 480, Software Version 1.5, Roche Diagnostics). All data were normalized to the previously validated internal housekeeping gene 30,31, ribosomal protein, large, P0 (*RPLP0*) using following primers: Forward 5′-TCTACAACCCTGAAGTGCTTGAT-3′ and Reverse 5′-CAATCTGCAGACAGACACTGG-3′. The reaction was performed under the following conditions: 95°C for 15 min., followed by 45 cycles of 95°C for 30 sec., 1 min. at 60°C; with the generation of the melting curve being performed at the end starting with 95°C for 5 sec. and following with increasing the temperature from 65 to 97°C with a heating rate of 2.5°C/sec. C_p_ values of each sample were normalized to the mean C_p_ value of the reference gene (α2A-AR/RPLP0 ratio = C_p_(α2A-AR)/C_p_ (reference gene)). The geometric mean of relative gene expression in a group of individuals with the wt genotype of the rs553668 polymorphism of α2A-AR gene was set to 1.

### Cell lines

All experiments were performed on HOS cell lines (ATTC CRL-1543) grown on DMEM and supplemented with 10% FBS (Gibco, Paisely, UK), 1% L-glutamate (Gibco) and 1% Antibiotic/Antimycotic (Gibco). Cell lines were passaged on a regular basis, as and when 80–90% confluence was achieved. No more than 20 passages were performed with a single cell line.

### Plasmid preparation and luciferase assays

Luciferase reporter assays were performed with pGl3-basic plasmids containing a 3′ UTR region of α2A-AR downstream of the *luc* gene and an α2A-AR promoter region upstream of the *luc* gene. All primers were designed based on the ENSEMBL genomic sequence of the α2A-AR gene (ENST00000280155). All PCRs were performed with a HiFi Polymerase Kit (QIAgene, Hilden, Germany). First, an additional polyclonal site containing *Eco*R1 and two *Ahd1* restriction sites followed by another *Eco*R1 site was inserted into the *Xba*1 site (GGAATTCGACCTTGAGTCGACAGATGGTCGAATTC) downstream of the *luc* gene to facilitate *ADRA2A* 3′ UTR cloning. Next, ATAAGATCTTGTTCGGAGATAGGAGAAGGC forward (containing a *Bgl*2 restriction site) and TTAAAGCTTGAACGATCAGCTCTCCAGGA (containing a *Hind*3 restriction site) reverse primers were used to amplify the 5′ promoter region of α2A-AR.

After obtaining the pGl3-basic with the α2A-AR promoter region, the 3′ UTR of α2A-AR from a heterozygous individual (polymorphism A/G rs553668) was amplified using the following primers: GTAGACTCACGCTGACTGCAG forward and GAAACTGTACAGTTTGGCAGGC reverse 17. The PCR product was cloned into a TOPO TA PCR cloning vector (Invitrogen, Carlsbad, CA, USA). Colonies were selected according to A or G genotype to obtain both α2A-AR 3′ UTR alleles. The inserted PCR was excised from the TOPO TA 4.0 vector using *Eco*R1 and cloned downstream of the *luc* gene into the newly established *Eco*R1 restriction site. The final plasmid constructs were sequenced to verify the sequence identity and orientation upstream and downstream of the *luc* gene. GTCTTACCGGAAAACTCGAC forward and TGCATTCTAGTTGTGGTTTGTC reverse primers were used to produce forward- and reverse-sequenced clones downstream of the *luc* gene. RVprimer3 CTAGCAAAATAGGCTGTCCC and GLprimer2 CTTTATGTTTTTGGCGTCTTCCA primers were used to produce forward- and reverse-sequence clones upstream of the *luc* gene. Sequencing was performed with the DTCS quick sequencing kit (Beckman Coulter, High Wycombe, UK) and reactions were separated on a GeXP Genomic Analyser (Beckman Coulter) in accordance with the manufacturer’s instructions.

Functional assays were carried out by plating cells at a density of 35,000 cells/well in 24-well tissue culture plates. After 24 hrs, the cells were transfected in six replicates with a mixture of Fugene HD transfection reagents (Roche Diagnostics, Mannheim, Germany) at a ratio (ml reagent:ml DNA) of 3:2, DMEM and 475 ng DNA/well and an additional 25 ng/well of a pRL-TK control reporter vector. Cells were harvested 48 hrs post transfection, and the luciferase assay performed with a Dual-Luciferase Reporter Assay System (Promega, Madison, WI, USA). Luminescence was measured using a BIO-TEK Synergy HT multidetection microplate reader (Fisher Scientific, Pittsburgh, PA, USA).

## Subjects

We evaluated 661 Slovenian people who were referred to the outpatient departments of the University Medical Centre in Ljubljana, the General and Teaching Hospital in Celje or the University Medical Centre Maribor for BMD measurement. BMD measurements at the lumbar spine (L2-L4) BMD-ls, total hip BMD-hip and femoral neck BMD-fn were performed by dual-energy X-ray absorptiometry (QDR-4500; Hologic, Inc., Waltham, MA, USA) in Ljubljana, Celje and Maribor. A cross-calibration study of the precision of measurements between the centres had previously been performed and a correction factor was not considered necessary. Each patient was examined clinically and routine biochemical tests were performed to exclude systemic and metabolic bone diseases other than primary OP. Subjects who had previously taken any drug known to influence bone metabolism were excluded from the study. Biochemical markers of bone turnover were measured in subgroups of subjects. Blood samples were collected between 8:00 a.m. and 10:00 a.m. after an overnight fast. The plasma and serum samples were analysed in a routine laboratory using standard procedures as outlined by the manufacturers. Osteocalcin (OC) in heparinized plasma was measured by means of a solid-phase, two-site chemiluminescent enzyme-labelled immunometric assay (Immulite Osteocalcin, Diagnostic Product Corporation, Los Angeles, CA, USA). Serum bone alkaline phosphatase (sBALP) was measured by radioimmunoassay (Tandem-R Ostase, Beckman Coulter, Fullerton, CA, USA). Serum C-terminal cross-linking telopeptides of type I collagen (CTX), osteoprotegerin and cathepsin K were measured by enzyme immunoassay (Serum CrossLaps ELISA, Nordic Bioscience Diagnostics A/S, Herlev, Denmark, Osteoprotegerin ELISA, Biomedica, Vienna, Austria, and Cathepsin K ELISA, Biomedica, respectively). The study was approved by the Ethical Committee of the Republic of Slovenia and written informed consent was obtained from each patient participating in the study.

### Genotyping on blood samples

DNA was isolated from peripheral blood leucocytes using a Flexigene kit (QIAgene) in accordance with the manufacturer’s protocol. Genotyping was performed with 5× HOT FIREPol® Probe qPCR Mix Plus (Solis BioDyne, Tartu, Estonia) and 10 ng of genomic DNA and the TaqMan genotyping assays C__996424_20 for rs553668 and C___7611979_10 for rs1800544 (both Applied Biosystems) respectively, in line with the manufacturer’s protocol. Allelic discrimination was performed on LightCycler 480® (Roche Diagnostics, Switzerland) employing the following protocol: 1 cycle of 15 min. at 95°C, followed by 45 cycles at 95°C for 15 sec., 60°C for 60 sec. and 4 min. at 40°C. An end-point genotyping program was used to call genotypes automatically.

### Statistical analysis and bioinformatics

Comparisons between genotype, and allele frequencies in subject subgroups were performed with a chi-squared test or Fisher’s exact probability test. The degree of linkage disequilibrium (LD), denoted as *D*’ and *r*^2^, between both SNPs was calculated using EMLD software (Qiqing Huang, University of Texas, Houston, TX; https://cge.mdanderson.org/∽qhuang/Software/pub.htm). The distribution of variables was assessed using the Shapiro–Wilk test and logarithmic transformation was used for variables that did not meet the normality assumption. The effects of genotypes, and alleles on BMD and biochemical markers of bone turnover were evaluated by means of the general linear model (GLM) adjusted for age, sex and body mass index (BMI) in all study subgroups. An LSD *post hoc* adjustment for multiple comparisons was used to find the differences within the genotype subgroups. The effects of the studied alleles on fracture risk were examined by logistic regression analysis in the group of all participants and, subsequently, in subgroups of elderly people, men and postmenopausal women. The model included the studied alleles (one or both alleles *versus* no allele under study), age (in years), BMI (in kg/m^2^) and femoral neck BMD (in g/cm^2^). The significance threshold was set at 0.05. SPSS Statistics version 21 (IBM, Chicago, IL, USA) was used for all statistical analyses. The power of our study was 61% to detect a 0.080 g/cm^2^ difference in BMD between the genotype and haplotype subgroups and 69.3–98.2% to discover a significant difference in the odds ratio of alleles between fracture patients and the no fracture controls 32.

The association of α2A-AR genotypes with α2A-AR gene expression in the study group as a whole was evaluated using the anova test once the normality of distribution had been confirmed.

TargetScan 6.2 (Whitehead Institute, Cambridge, MA, USA) 33 and miRANDA programs 34 were used to search for conserved miRNA sites in the region of the 1780G>A polymorphism. The Unafold 4.6 35 program was used to predict the secondary structure of RNA and estimate the structure’s stability by calculation of its free energy.

## Results

### Immunohistochemistry

**Figure 1:**
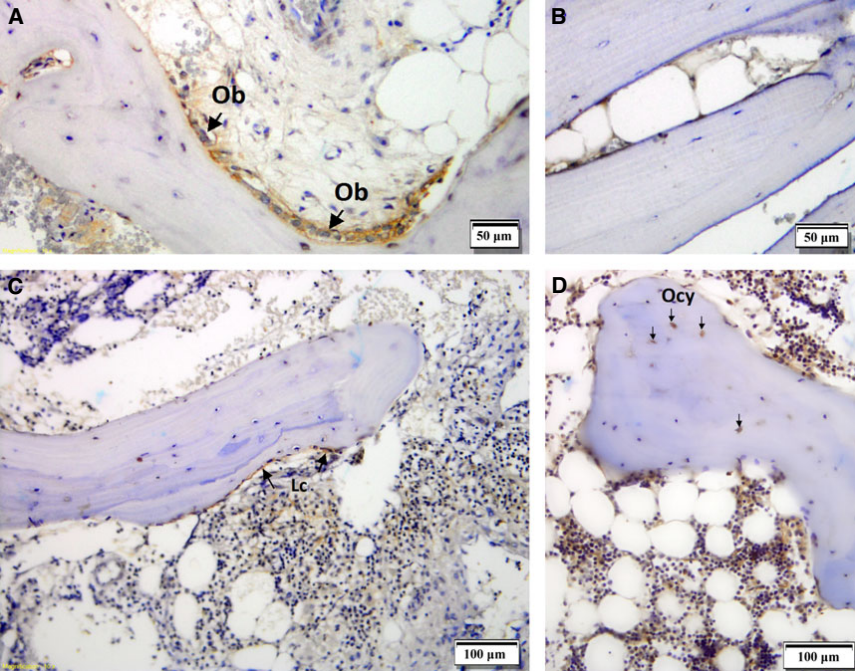
Immunolocalization of α2A-AR in human bone samples from an OP patient (A and C), OA patient (B) and an autopsy case (D). Positive α2A-AR staining was observed in cuboidal shaped osteoblasts (Ob) and lining cells (Lc). No staining was observed in any of the control slides where no α2A-AR antibody was applied (negative control, data not shown).

**Figure 2:**
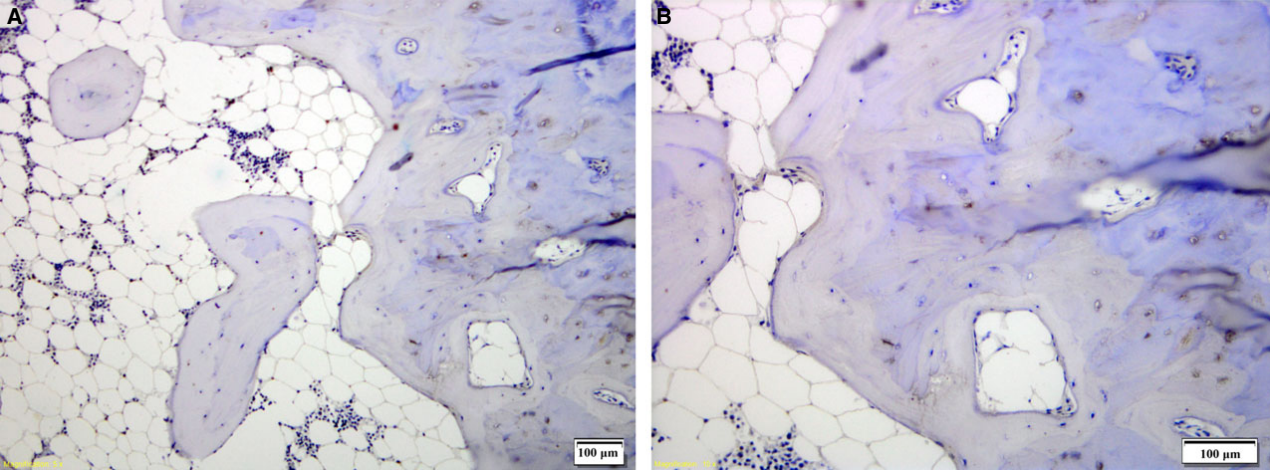
A cross-section of the human femoral head bone from the autopsy case shows no α2A-AR staining of osteocytes in the cortical and trabecular bone (A and B). No staining was observed in any of the control slides where no α2A-AR antibody was applied (negative control, data not shown).

To understand whether α2A-AR could be involved in neuro-endocrine signalling, its presence in human bones was investigated using immunohistochemistry. In addition, we has been suggested that since ARKO knockout mice show higher bone phenotype, we might see the difference in α2A-AR distribution in bones of different density. As a result of the qualitative and at best semi-quantitaive nature of immunohistochemistry, we used the method only to investigate α2A-AR localization within bone sample. Immunohistochemistry was performed on the four bone samples showing different α2A-AR expression according to real-time PCR (see Table1 for average α2A-AR mRNA expression), the highest (OP patient – relative expression of 1.7388), an intermediate (OA patient – relative expression 1.0407) and the lowest (autopsy participant and one OP patient – relative expression 0.7752 and 0.8840 respectively). This was performed to understand whether the difference in α2A-AR mRNA expression was because of different cells expressing α2A-AR or because of difference in numbers and/or expression of α2A-AR on the cells carrying the receptor. The results show that α2A-AR is present on osteoblasts and lining cells but not osteocytes (Fig.1A and C) regardless of the twofold difference in α2A-AR mRNA expression level between these two samples. The most intensive staining was observed in the sample from the OP patient with the highest α2A-AR gene expression, where cuboidal shaped osteoblasts with intensive α2A-AR staining were present (Fig.1A). The OP sample with the lowest α2A-AR gene expression (Fig.1C) also showed intensive staining in lining cell and osteoblasts. Both OP samples contained α2A-AR negative osteocytes (Fig.1A and C). OA and autopsy sample (Fig.1B and D) did not include any cuboidal shaped osteoblasts. Individual osteocytes were positive for α2A-AR in trabecular bone of autopsy sample (Fig.1D). To examine α2A-AR distribution in a denser bone, a cross-section of the femoral head from the autopsy participant was performed (Fig.2). Negative α2A-AR staining for osteocytes in the cortical and trabecular bones in the cross-section of the autopsy sample was observed (Fig.2A and B). All negative controls performed did not show unspecific binding of secondary antibody. Results suggest that α2A-AR is predominantly expressed by osteoblasts and lining cells. Some osteocytes of trabecular bone may show α2A-AR expression but not osteocytes of cortical bones such as femoral head. Level of expression does not appear to be because of the different cells expressing α2A-AR, but rather because of the number of osteoblasts and lining cells in samples in comparison to other cells and/or α2A-AR mRNA expression level of these two cell lineages.

### Gene expression association study

**Figure 3:**
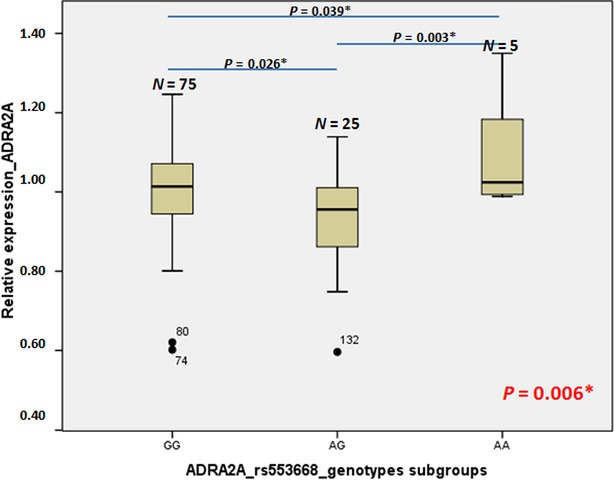
Box plots depicting relative α2A-AR gene expression according to genotype in bone; expression for the reference genotype (associated with low α2A-AR gene expression) is set to 1. Numbers above the boxes indicate the number of individuals with that genotype. The bottom and top of each box indicate the 25th and 75th percentiles, respectively, while the line inside the box is the mean value. Whiskers represent the smallest and the values that are not outliers. Circles with the code of the sample indicate outliers. The *P*-value in red represents the anova statistical result for differences in α2A-AR gene expression according to α2A-AR genotypes subgroups, while *P*-values in black represent the LSD post hoc testing between each of two genotype subgroups. *P*-values with asterisks are statistically significant (below 0.05).

Gene expression study was performed to further support results that α2A-AR is expressed in bone. Second, we tested the association of rs553668 and rs1800544 SNPs to find genetic factors that influence α2A-AR expression, useful for further genetic analysis. Of 140 samples, 113 samples tested positive for the presence of α2A-AR mRNA using real-time PCR (Table1), supporting above obtained immunohistochemistry result showing α2A-AR is indeed present in bone tissue. Next, association between amount of α2A-AR transcript and rs553668 and rs1800544 was investigated to test functionality of both SNPs in α2A-AR gene locus. Significant difference in the expression of α2A-AR and presence of the rs553668 was observed (Fig.3) when performing univariate statistical model adjusted for age, sex, height, weight and disease (*P* = 0.003). Statistical significant result was obtained also when no adjustment was made with slightly higher *P*-value of 0.006, showing that co-variables do not have much influence on α2A-AR gene expression. The highest expression was shown in AA genotype carriers, which was significantly different from the expression in GG genotype carriers; namely, by 10%. Heterozygote carriers showed the lowest expression (lower by 15% and 6% compared to AA and GG genotype carriers, respectively, Fig.3). The number of AA genotype samples was too small (*N* = 5) to draw complete conclusions. To clarify the effect of the A allele on gene expression, we continued with a Luciferase assay. Importantly no statistically significant difference in α2A-AR gene expression between male and female was observed. No statistically significant difference in α2A-AR gene expression was observed between disease groups although the OP group showed slightly but not statistically significant difference in comparison to OA or control group (Table1).

The association between the gene expression of the α2A-AR and rs1800544 SNP was not significant in this study group (*P* = 0.644).

### Luciferase assay and bioinformatics

**Figure 4:**
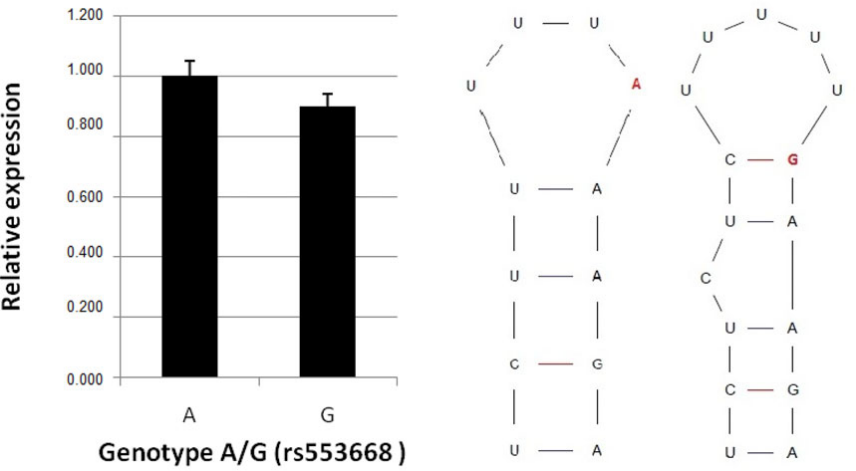
Left: relative expression of pOM-A and pOM-G plasmids. Right: Secondary structures formed by mRNA containing A (left structure) and G (right structure).

Luciferase reporter assay was performed to further support the evidence of rs553668 SNP functionality in osteoblasts. We constructed two novel plasmids containing 3′ UTR region of the α2A-AR cloned behind the luciferase reporter gene in pGl3-Basic to study the rs553668 SNP influence on mRNA stability. Each plasmid contained either G or A variant of rs553668 SNP. Transient transfections with two different plasmids were performed for an mRNA stability evaluation. Forty-eight hours post transfection, Renilla and firefly luciferase activities were measured and expressed as a ratio of firefly to Renilla activity. The relative ratio of luciferase activity was 1.000 and 0.896 in the A and G genotypes respectively (Fig.4), with the difference between the ratios being statistically significant (*P* < 0.012). This result supports the *in vivo* measurement of α2A-AR gene expression, where lower amount of mRNA was observed in individuals carrying G variant of rs553668 SNP.

A section of 3′ UTR was analysed using TargetScanHuman and miRANDA programs to evaluate if miRNA binding might play a role in α2A-AR mRNA stability. No binding site for miRNA was found in the vicinity of the polymorphic site. Next, the influence of rs553668 SNP on α2A-AR mRNA secondary structure formation was evaluated. Two distinct but equally stable (ΔG = −2.4 kcal/mol) structures were noted (Fig.4) suggesting the possibility that rs553668 SNP could function through difference in secondary structure of 3′ UTR of α2A-AR mRNA.

### Genotype association study

To investigate the possible association of α2A-AR gene locus with BMD and other biochemical turnover markers and to identify individuals at possible higher risk for OP genotype association study was performed. Above confirmed functional SNP rs553668 and SNP rs1800544 both in LD, were used. Rs1800544 did not show functionality on the level of α2A-AR mRNA. It was, nevertheless, included in the genetic association study because it was identified as a tag SNP of the α2A-AR gene locus, with a frequency favourable for genetic association study. The frequencies of rs553668 and rs1800544 SNPs in the α2A-AR gene were determined by screening DNA samples from 661 Slovenian individuals, divided into four subgroups with characteristics as presented in Table2.

**Table 2:** Anthropometric characteristics of the study population for α2A-AR genotyping

	Premenopausal women (*N* = 53)	Postmenopausal women (*N* = 429)	Elderly men (*N* = 108)	OP patients (*N* = 71)
Female/male	53/0	429/0	0/108	54/17
Age (years)	45.7 ± 4.8	61.9 ± 8.5	67 ± 6	78 ± 7
Body mass index (kg/m^2^)	24.5 ± 4.2	28.2 ± 5.0	28.0 ± 3.5	26.1 ± 3.6
Hip BMD (g/cm^2^)	0.924 ± 0.089	0.914 ± 0.136	0.809 ± 0.151	0.597 ± 0.097
T score (hip)	−0.2 ± 0.7	−0.4 ± 1.0	−1.2 ± 1.1	−2.6 ± 0.9
Femoral neck BMD (g/cm^2^)	0.783 ± 0.076	0.763 ± 0.120	1.021 ± 0.160	0.678 ± 0.125
T score (FN)	−0.7 ± 0.7	−1.0 ± 1.0	−0.3 ± 1.0	−2.4 ± 0.9
Lumbar spine L2-L4 BMD (g/cm^2^)	1.003 ± 0.117	0.974 ± 0.137	1.061 ± 0.189	0.862 ± 0.174
T score (LS)	−0.6 ± 1.0	−0.8 ± 1.2	−0.4 ± 1.6	−1.8 ± 1.6

Values are mean ± SD.

BMD, bone mineral density; FN, femoral neck; LS, lumbar spine.

The distributions of genotypes for both SNPs are shown in Table3. The genotype frequencies for each polymorphism were consistent with the Hardy–Weinberg distribution (*P* > 0.05) as a whole and in all subgroups. There were no significant differences in both SNPs genotypes between subgroups (*P* > 0.05). The genotype frequencies were similar to those in the HapMap for rs553668, while rs1800544 showed a higher frequency of the minor genotype GG (1.4–5.8% *versus* 0%) in our study groups compared to PubMed data for the European population (NCBI dbSNP Build 141, http://www.ncbi.nlm.nih.gov/projects/SNP/snp_ref.cgi?rs=rs1800544). Both SNPs were in LD, with *r*^2^ = 0.56 and *D*’ = 0.986.

**Table 3:** Results of A>G (rs553668) and C>G (rs1800544) genotyping analysis and their association with BMD and biochemical markers of bone turnover in subgroups of subjects

	A>G (%)	C>G (%)	A>G (rs553668)	C>G (rs1800544)
Premenopausal women	G/G 37 (69.8)	C/C 28 (52.8)	Cathepsin K (*P* = 0.014)	NS
	G/A 16 (30.2)	C/G 22 (41.5)	AG: 18.3 ± 19.2 pmol/l	
	A/A 0 (0)	G/G 3 (5.7)	GG: 3.5 ± 19.1 pmol/l	
Postmenopausal women	G/G 307 (71.6)	C/C 257 (59.9)	NS	BMD-ls (*P* = 0.027)
	G/A 114 (26.6)	C/G 147 (34.3)		GG: 0.802 ± 0.155 g/cm^2^
	A/A 8 (1.9)	G/G 25 (5.8)		GC: 0.880 ± 0.157 g/cm^2^
Elderly men	G/G 87 (80.6)	C/C 79 (73.1)	NS	sCTX (*P* = 0.012)
	G/A 20 (18.5)	C/G 26 (24.1)		GC: 3305 ± 988 pmol/l
	A/A 1 (0.9)	G/G 3 (2.8)		CC: 1689 ± 949 pmol/l
OP patients	G/G 60 (84.5)	C/C 49 (69.0)	pOC (*P* = 0.009)	sBALP (*P* = 0.053)
	G/A 10 (14.1)	C/G 21 (29.6)	AG: 11.8 ± 5.2 μg/l	GC: 9.6 ± 2.9 μg/l
	A/A 1 (1.4)	G/G 1 (1.4)	GG: 5.4 ± 5.2 μg/l	CC: 7.2 ± 2.9 μg/l

Values are number of frequencies (percentages) and mean ± SD. Differences were obtained using general linear model.

NS, *P* > 0.05.

BMD-ls, lumbar spine bone mineral density; sCTX, serum C-terminal crosslinking telopeptides of type I collagen; pOC, plasma osteocalcin; sBALP, serum bone alkaline phosphatase concentration; cathepsin K, serum cathepsin K concentration.

The results of GLM analysis are presented in Table3. Significant correlations were observed between α2A-AR gene locus and BMD at lumbar spine in postmenopausal women and markers of bone turnover sCTX in elderly men, cathepsin K in premenopausal women, pOC and borderline with sBALP in OP patients.

## Discussion

In this study, the further evidence for involvement of α2A-AR signalling in neuro-endocrine regulation of bone remodelling is presented. This study is the first to provide evidence on the level of human bone tissue, using immunohistochemical, and functional characterization of genetic polymorphisms influence on the level of α2A-AR transcript. The research shows that not only β2-AR is involved in neurological signalization but also that α2A-AR will have to be considered in the future.

First evidence of α2A-AR involvement in neuro-endocirne regulation of bone remodelling was obtained by identification of bone cells possessing α2A-AR. The results show that α2A-AR is present on osteoblasts and lining cells (Fig.1A and C). The receptor may be present on individual osteocytes of trabecular bone (Fig.1B and D), but we have not detected the receptor on osteocytes of denser cortical bone (Fig.2A and B). This result is in agreement with a recent report on ARKO knockout mice that showed increased bone mass 13. To further support the data on α2A-AR expression in human osteoblasts and lining cells, real-time PCR was performed to detect gene transcript in bones. Results obtained on 140 bones samples show presence of α2A-AR mRNA transcript in 105, supporting conclusion that α2A-AR receptor is expressed in bone remodelling cells – osteoblasts and lining cells. Because histological results did not contain osteoclasts the conclusion whether they contain α2A-AR was not possible. To provide further evidence of α2A-AR association with bone metabolism, genetic association study was performed. Prior to that, role of two SNPs rs553668 and rs1800544 was investigated to provide evidence of their functionality. The results on rs553668 SNP show that variant G appears to have destabilizing effect on α2A-AR mRNA probably through formation of different secondary structure and not through miRNA mediated degradation process as there appear to be no miRNA binding sites in vicinity of polymorphic site. The rs1800544 did not correlate with α2A-AR mRNA expression, therefore no further luciferase reporter tests were carried out. Subsequent genetic association study using both SNPs showed significant association of α2A-AR gene locus and BMD and markers that reflect bone metabolism. The result supports the conclusion that α2A-AR present on oseoblasts may be actively involved in bone remodellation. In support of these results, the same extensive chromosome 10q24-q26 location, containing multiple genes, has previously been shown to be associated with OP and BMD 1.

The precise pathway of α2A-AR action is unknown. Our results suggest action through osteoblasts and/or lining cells because they carry the receptor. Several mechanisms of action might be envisaged. Signalling may either result in apoptosis and/or inhibition of osteoblast proliferation or in stimulation of osteoclast differentiation, tipping the bone remodelling balance in favour of bone resorption. Evidence from the study on ARKO mice shows latter might be more plausible, since decreased expression of RANKL was observed in mutant mice 13. Similar findings were observed on β2-AR receptors. Osteoblasts having viable β2-AR receptor were able to stimulate osteoclast formation in contrast to β2-AR null osteoblast. The signalling was mediated through ATF4 phosphorylation and the main ligand for β2-AR was leptin 6,8. Authors also claimed that β2-AR is the only AR expressed by osteoblast 6,8 which would be in contrast to our and other 13 results. However, it appears from their manuscript that expression of α2A, α2B and α2C-AR receptors was not investigated on culture of primary mouse osteoblasts 6,8, therefore, our study is the first to investigate and prove their presence in human bones. The finding of other ARs on osteoblasts is interesting because both knockout mice (ARKO and β2-AR) produce similar high bone phenotype. The result points to possible interaction of ARs. One possible explanation of why similar bone phenotypes were obtained when knocking out either of ARs might involve well known process of ARs dimerization 36. The other more indirect explanation may involve receptor cross-talk that showed how β2-AR stimulation resulted in increased α2A-AR internalization on the same neuron cell 37 or its desenzitation by recruiting GRK protein to plasma membrane 38. Although the precise mechanism of action on osteoblasts and lining cells remains to be elucidated our finding of new ARs in bone might have possible clinical implications. The use of beta blockers has for some time been considered as a treatment option for bone fracture healing and treatment of OP 39. Because signalling through α2-AR appears to have similar effect on bone as β2-AR, antagonists and agonists of α2-AR should be explored for their beneficial and detrimental effect on bone.

In conclusion, the study presents further evidence based on human samples that α2A-AR receptors are important in osteoblast neuro-endocrine signalling, bringing a new player repetition along with already known β2-AR. Although the precise pathway of events remains to be elucidated, it looks like the neurological signal of bone remodelling is mediated through osteoblasts and/or lining cells which seem to express most of the α2A-AR of bone remodelling cells. The study also shows that functional polymorphism in 3′ UTR of α2A-AR gene affects its mRNA stability and is associated with bone turnover markers.
